# Uncommon Bilateral Carotid Artery Dissection in a Farmer: The Perils of Heavy Weight Lifting

**DOI:** 10.7759/cureus.45797

**Published:** 2023-09-22

**Authors:** Asha Sahu, Tanushree Chawla, Tamanna, Jai P Sharma, Vinay Goyal

**Affiliations:** 1 Department of Neuroscience, Medanta The Medicity Hospital, Gurugram, IND; 2 Department of Radiology, Medanta The Medicity Hospital, Gurugram, IND

**Keywords:** farmer, non-traumatic carotid artery dissection, weight lifting, life threatening, quadriparesis, acute neck pain, young-onset stroke

## Abstract

Bilateral internal carotid artery (ICA) dissection on heavy weight lifting is a very rare cause of stroke in young patients. Arterial dissection is due to a tear in the intima and internal elastic lamina which leads to extravasation of blood into the media and subintimal plane. Clinical diagnosis of carotid artery dissection is difficult with common clinical presentations like headache and neck pain. Here we present a case of a 40-year-old young man who presented to us (tertiary referral center) with headache and quadriparesis. MRI brain showed multiple acute infarcts in bilateral centrum semiovale extending up to frontal periventricular white matter with few tiny foci in bilateral medial temporal and left gangliocapsular regions and CT cerebral angiography showed bilateral ICA dissection. He was treated with low molecular weight heparin and was discharged for follow-up with regular physiotherapy.

## Introduction

Bilateral internal carotid artery (ICA) dissection is itself a rare condition with an incidence of up to five cases per 100,000 with a slight male predilection of 55% [1,2]. The patient initially presents with symptoms of acute ischemic stroke. Internal carotid artery (ICA) dissection is a life-threatening emergency and it has to be treated to prevent a catastrophic outcome. ICA dissection may be missed in patients with minor strokes due to a lack of symptoms specific to ICA dissection, as only 10% of patients show immediate symptoms of ICA dissection. Arterial dissection is due to tears in the intima and internal elastic lamina which leads to extravasation of blood into the media and subintimal plane [3]. Blood enters the layers of the arterial wall and results in stenosis or aneurysmal dilatation [4].

## Case presentation

A 40-year-old gentleman with a history of lifting heavy stacks of sugarcane (approximately 100 stacks) 2 days back while working at his farm, presented with sudden onset holocranial headache with giddiness and transient blurring of vision for 2 days. He was found to have high blood pressure (180/100 mmHg). He was managed conservatively with antihypertensives (details were not available). The next day, he developed left hemiparesis with a further fall in his sensorium. At presentation to this hospital, he was conscious with quadriparesis, power was MRC (Medical Research Council) grade 4/5 on the right side and 2/5 on the left side at all joints, with plantar bilaterally extensor.

**Figure 1:**
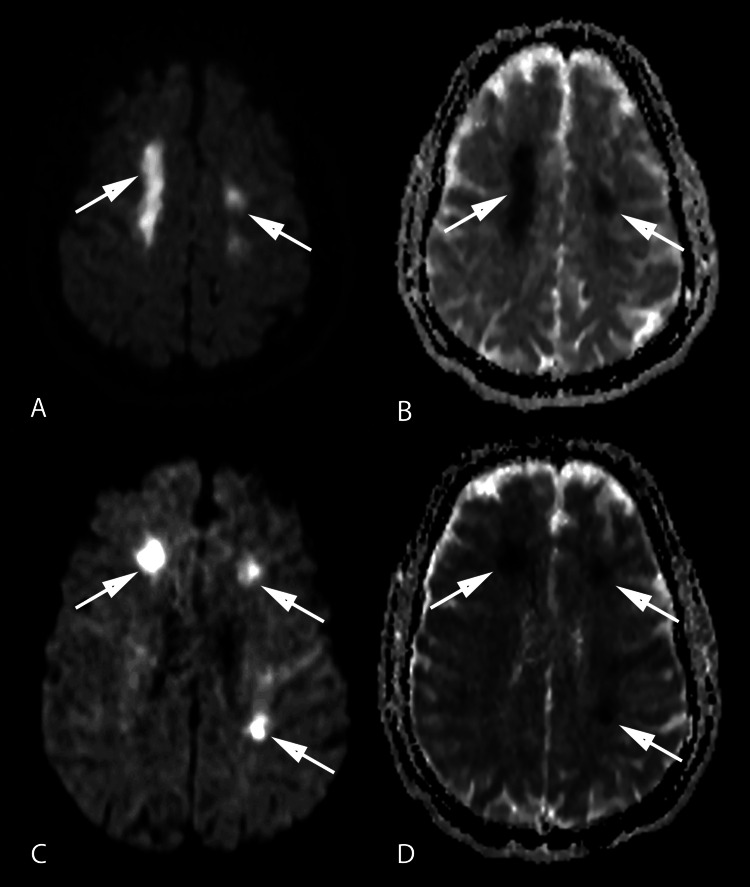
MRI Brain showing acute ischemic infarcts A and C: Axial DWI images of the brain showing multiple areas of diffusion restriction in bilateral centrum semiovale extending up to frontal periventricular white matter; B and D: Axial ADC images of the brain showing areas of low ADC values in bilateral centrum semiovale and periventricular white matter suggesting acute infarcts MRI = Magnetic Resonance Imaging; DWI = Diffusion-Weighted Imaging; ADC = Apparent Diffusion Coefficient

MRI brain showed multiple acute ischemic infarcts in bilateral centrum semiovale extending up to frontal periventricular white matter with few tiny foci in bilateral medial temporal and left gangliocapsular regions (Figures 1A-1D).

**Figure 2:**
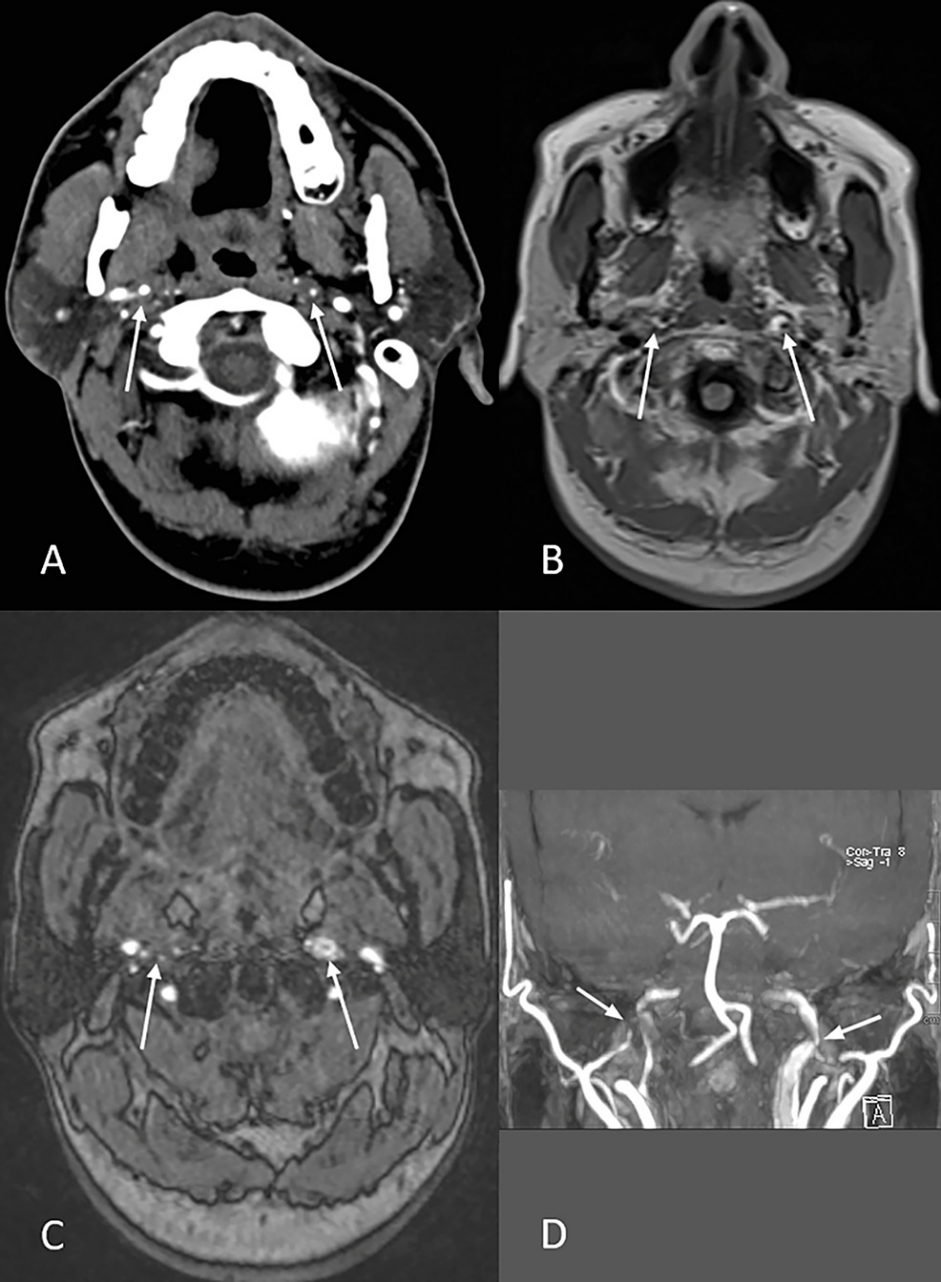
CT and MR angiograms showing Internal carotid artery dissection A: Axial CT image of the brain showing ill-defined circumferential hypodensity eccentrically surrounding bilateral cervical ICA; B: Axial T1W FLAIR image of the brain showing curvilinear hyperintensity at skull base around distal-most part of bilateral ICA just below the level of intrapetrous segments resulting in moderate to significant luminal stenosis of the lumen of bilateral ICA; C: Axial TOF image of the brain showing heterogeneously hypointense signal around bilateral ICA causing mild to moderate luminal stenosis; D: Coronal MR angiogram MIP image showing heterogeneously hyperintense signal around bilateral ICA. CT = Computed Tomography; MR = Magnetic Resonance; TOF = Time of Flight; ICA = Internal Carotid Artery; MIP = Maximum Intensity Projection; T1W FLAIR = T1-Weighted Fluid-Attenuated Inversion Recovery

CT cerebral angiography showed ill-defined circumferential hypodensity eccentrically surrounding bilateral cervical ICA in the mid and distal segment resulting in significant stenosis and irregularity reaching up to the skull base (Figures 2A-2D).

His other routine stroke workup including electrocardiogram, 2D echocardiography, transesophageal echocardiography, serum homocysteine levels, connective tissue (protein C, protein S, antithrombin III activity, Antinuclear Antibody (ANA)/Antinuclear Factor​​​​​​​ (ANF), Indirect Fluorescent Antibody​​​​​​​ (IFA), Erythrocyte Sedimentation Rate​​​​​​​ (ESR), C-Reactive Protein​​​​​​​ (CRP), phospholipid antibodies panel IgG, IgM,) was normal. He was treated with low molecular weight heparin. On day 5, he showed clinical improvement and was discharged for follow-up with regular physiotherapy.

## Discussion

Bilateral internal carotid artery dissection in young patients is extremely rare [5,6]. Dissection is more common in the internal carotid artery with a higher incidence in the extracranial segment of the ICA, approximately 2-3 cm superior to the common carotid bifurcation, and evidence shows that this site accounts for about 2.5% of all first strokes [6]. ICA dissection can be spontaneous, secondary to trauma or iatrogenic causes. Spontaneous ICA dissection is more common in patients with congenital connective tissue disorder and fibromuscular dysplasia. Risk factors of ICA dissection are hypertension, smoking, hyperhomocysteinemia, connective tissue disorders, migraine, and smoking [7]. The pathophysiology behind arterial dissection is the destruction of smooth muscles and elastic fibers in the tunica media causing blood to enter between the layers of the arterial wall, creating a false lumen which is seen as an intimal flap or double lumen on angiography [7].

MRI has replaced conventional angiography for diagnosing ICA dissection due to the improved resolution of recent MRI machines and the non-invasive nature of the scan. MRI also helps in visualizing the intra-mural hematoma. Further, it can also be diagnosed incidentally on cerebral or neck angiogram. However, the pathognomic features of imaging are seen in only 10% of patients. More frequently detected findings on imaging are irregularity of the vessel wall as seen in this patient and signs of stenosis and occlusion about 2 cm distal to the carotid bulb [8].

The appropriate MRI protocol includes a magnetic resonance angiogram (MRA) of the neck to evaluate the lumen and axial fat-suppressed T1-weighted images to evaluate for subacute intramural haematoma. However, when the MRI protocol is not tailored accordingly, subpetrous internal carotid artery dissection with intramural haematoma can still be detected on routine brain MRI sequences [9]. Double inversion recovery - black-blood imaging (3T DIR-BBI) offers excellent visualization of the lumen, wall, and periadventitia so it is useful in the evaluation of acute cervical artery dissection. This technique could be used either for primary diagnosis or as an adjunct in difficult or inconclusive cases. Its high spatial and contrast resolution offers a mode of further exploration into the pathogenesis of cervical artery dissection [10]. MRI black blood imaging was not performed in our case as it is not being done routinely at our center. We have diagnosed carotid artery dissection in our case with the help of MR angiography and CT angiography.

Our patient did not show any clinical features suggesting connective tissue disorder. Traumatic events like road traffic accidents, and sports-related trauma to the head and neck can cause ICA dissection, or mechanical stress like heavy weight lifting, strong coughing or vomiting can be an important contributing factor in ICA dissection [11].

A case of cough-induced extracranial internal carotid artery dissection was reported by Mei-Ling Sharon Tai et al. in 2020 [11]. A patient with a history of intractable vomiting presenting with spontaneous dissections of the bilateral internal carotid and vertebral arteries was reported by Echefu G et al in 2022 [12]. Due to Eagle syndrome with Ehlers-Danlos syndrome was reported by Ikenouchi H et al. [13] and bilateral carotid artery dissection with Foix-Chavany-Marie syndrome was reported by Singh R et al. [14].

This is an extremely rare incidence where routine heavy weight lifting during farming has led to bilateral carotid artery dissection. This suggests that people lifting heavy weights are at risk of ICA dissection and should be advised to carry weights in smaller batches and should keep some rest period between lifting weights.

## Conclusions

In conclusion, this is a rare case of stroke with bilateral ICA dissection in a young farmer. We would like to emphasize that ICA dissection, though rare, is an important cause of stroke in the young, which is often missed. History of farm-related heavy weight lifting and neck pain shall be carefully elicited and must raise a suspicion of dissection. Heavy weight bearing is an important trigger factor for developing carotid arterial dissection.
